# Effect of different routes of magnesium sulfate administration combined with quadratus lumborum block on postoperative analgesia and recovery quality in patients undergoing laparoscopic total hysterectomy: a prospective, randomized controlled trial

**DOI:** 10.3389/fmed.2025.1600630

**Published:** 2025-08-29

**Authors:** Wen-xiu Wang, Li Zhang, Shu-zhi Zhou, Xin Ran, Jie Zhang

**Affiliations:** ^1^Department of Anesthesiology, Zigong First People Hospital, Zigong, Sichuan, China; ^2^Department of Anesthesiology, Ya'an People Hospital, Ya'an, Sichuan, China; ^3^Department of Pain Medicine, Zigong Fourth People Hospital, Zigong, Sichuan, China

**Keywords:** quadratus lumborum block, magnesium sulphate, uterine surgery, postoperative pain, quality of recovery

## Abstract

**Objective:**

To investigate the effects of different ways of magnesium sulfate injection combined with ultrasound-guided quadratus lumborum block (QLB) on postoperative analgesia and recovery quality of patients undergoing laparoscopic total hysterectomy under general anesthesia.

**Method:**

A total of 88 patients who underwent laparoscopic hysterectomy in Ya’an people’s Hospital from June, 2020 to February, 2022, aged from 18 to 65, were randomly divided into three groups: control group (group A, *n* = 29), local magnesium sulfate group (group B, *n* = 29) and intravenous magnesium sulfate group (Group C, *n* = 30) All patients were treated with general anesthesia and patient controlled intravenous analgesia (PCIA) after surgery. General information, visual analogue scale (VAS) at 2 h, 6 h, 12 h, 24 h, 48 h after surgery, dosage of sufentanil in 24 h after surgery, the duration of QLB analgesia (time of first pressing analgesic pump after surgery), 40-item quality of recovery score (QoR-40) before the surgery and on the 3d after surgery, hypotension, bradycardia, postoperative nausea and vomiting (PONV) at 1-3d after surgery, postoperative delirium (POD), skin pruritus, chills, lower limb movement block and other adverse reactions were observed and recorded.

**Result:**

Compared with group A, VAS score and postoperative sufentanil consumption were decreased in group B and group C, and the onset time of first postoperative analgesia request was prolonged (*p* < 0.05), while there was no statistically significant difference between group B and group C (*p* > 0.05). The QoR-40 score of group B and group C on 3d after surgery was higher than that of group A (*p* < 0.05), while there was no statistically significant difference between group B and group C (*p* > 0.05). The incidence of PONV in group B and group C was lower than that of group A, and the difference was statistically significant (*p* < 0.05), while there was no statistically significant difference between group B and group C (*p* > 0.05). No hypotension, bradycardia, POD, pruritus, chills, lower limb movement block were observed.

**Conclusion:**

Intravenous or local use of magnesium sulfate combined with QLB has similar postoperative analgesic effect and promoting postoperative recovery effect in patients undergoing laparoscopic hysterectomy, which can reduce the use of postoperative analgesic drugs, provide effective analgesia and improve the quality of postoperative recovery.

**Clinical trial registration:**

Chinese Clinical Trial Registry (https://www.chictr.org.cn/), identifier ChiCTR2200055978.

## 1 Introduction

Laparoscopic total hysterectomy which has the advantages of light trauma, quick recovery and beautiful incision has been widely used in gynecological diseases in recent years (1). However, due to the existence of abdominal wall incision and surgical trauma, moderate to severe pain is common after surgery, which may even affect the postoperative rehabilitation and prolonged hospital stay of patients (2, 3). Therefore, patients undergoing laparoscopic hysterectomy also need satisfactory postoperative analgesia. Effective postoperative analgesia can help patients recover quickly, reduce complications, shorten hospitalization time and reduce hospitalization costs. It also conforms to the concept of enhanced recovery after surgery (ERAS) (4). At present, there are many methods for postoperative analgesia in laparoscopic surgery. PCIA can provide accurate postoperative analgesia and has a wide range of clinical applications, but it has risks such as postoperative nausea and vomiting, dizziness, intestinal paralysis and even respiratory depression. Epidural analgesia technique has good analgesic effect, but it also has side effects such as nausea and vomiting, urine retention, hypotension, etc. Besides, the operation is relatively complicated, there are more complications related to puncture, and it will affect patients’ early ambulation activities (5–7). Transversus abdominis plane block (TAP) is another commonly used postoperative analgesia for abdominal surgery, but it also has limitations, such as shorter analgesia time, narrow block range, often limited to the lower abdomen, and poor visceral pain inhibition effect (8). The quadratus lumborum block (QLB) is a novel trunk nerve block technique which was first proposed by Blanco in 2007. It refers to the process of injecting local anesthetics around the quadratus lumborum muscle and allowing the liquid to process throughout the thoracolumbar fascia (TLF). It can block thoracolumbar nerves and produce effective analgesia, so it can be widely applied to perioperative analgesia of abdominal, hip and lower limb operations (9, 10). Currently, QLB is widely used in patients undergoing abdominal surgery, with strong analgesic effect and long duration, which can reduce the use of opioids and the occurrence of postoperative complications (11). Magnesium sulfate is a natural non-competitive N-methyl-D-aspartic acid (NMDA) receptor blocker, which can act on the NMDA receptor, reduce the sensitivity of the central nervous system to pain, reduce peripheral pain sensitising, and reduce the release of catecholamines and neurotransmitters, resulting in analgesic effects (12, 13). Studies have shown that magnesium sulfate can play an analgesic role by intravenous injection, intrathecal injection and local anesthesia adjuvant (14–16).

However, there are few reports on the use of different routes of magnesium sulfate injection combined with quadrat plane block for postoperative analgesia. This study observed the effects of magnesium sulfate injection combined with ultrasound-guided posterior quadrate block on postoperative pain and postoperative recovery quality of patients undergoing laparoscopic total hysterectomy under general anesthesia, so as to provide a safer and more effective analgesic program for clinical anesthesia.

## 2 Data and methods

### 2.1 General information

This study has passed the ethical review by the Ethics Committee of Ya ‘an Personnel Hospital (ethics approval no.: 2021-10) and completed the Chinese clinical trial registration (registration no.: ChiCTR2200055978). Patients who underwent laparoscopic hysterectomy with general anesthesia in the Department of Obstetrics and Gynecology of Ya ‘an People’s Hospital from June 31, 2020 to February 1, 2022.

were selected. Inclusion criteria: age 18-65;patients undergoing laparoscopic total hysterectomy; ASA I ~ III grade; no other serious complications, contraindications to surgery or distant metastasis of the tumor; body mass index (BMI) ranges from 18.5 to 28.0 kg/m^2^. Exclusion criteria: patients with peripheral diabetic lesions and contraindicated nerve block; people with a history of serious heart, liver, kidney and other organ diseases; long-term use of calcium channel blockers, hypermagnesemia; those who have a history of mental illness, severe hearing and vision impairment or other reasons that prevent them from communicating and visiting; patients and their families refused. Exit criteria: failure of nerve block; severe complications or death occurred during perioperative period; incomplete data collection; patients who change the operation method or open the abdomen midway. The patients were randomly divided into three groups. In group A, patients received saline (0.9%) infusion 50 mL in travenous within 15 min followed by 10 mL/h infusion maintenance dose till the end of surgery. Bilateral QLB block was performed at the same time, and 0.375% ropivacaine 20 mL + 2 mL normal saline was injected into each side. In Group B, patients received saline (0.9%) infusion 50 mL in travenous within 15 min followed by 10 mL/h infusion maintenance dose till the end of surgery. Bilateral QLB block was performed at the same time, and 0.375% ropivacaine 20 mL + 25% magnesium sulfate 2 mL (0.5 g) was injected into each side. In Group C, patients received magnesium sulfate 50 mL at a dose of 30 mg/kg within 15 min followed by 10 mg/(kg h) infusion maintenance dose till the end of surgery. At the same time, bilateral QLB block was performed, and 0.375% ropivacaine 20 mL + 2 mL normal saline was injected into each side.

### 2.2 Anesthesia method

On arrival to the operating room, an IV cannula 20G was inserted, and Ringer’s acetate solution was infused. A five-lead electrocardiogram, a pulse oximeter, and a noninvasive blood pressure cuff were applied. Then, midazolam 0.04 mg/kg and penehyclidine hydrochloride 0.01 mg/kg were injected intravenously, followed by bilateral QLB2 block. Next, general anesthesia was induced using propofol 2 mg/kg, sufentanil 0.3 μg/kg, and rocuronium 0.6 mg/kg to facilitate endotracheal intubation. The tidal volume was set at 8 ~ 10 mL/kg, and the ventilation frequency was set at 10 ~ 14 times/min. Sevoflurane was given 1–2% and remifentanil 0.1–0.3 μg/(kg·min) to maintain anesthesia, and rocuronium was added intermittently to maintain muscle relaxation. The ventilator parameters were adjusted to maintain PET CO_2_ at 30 ~ 40 mmHg. The pump dose of remifentanil was adjusted according to blood pressure, heart rate and BIS results, and appropriate fluid rehydration or vasoactive drugs were used to ensure that the fluctuation range of intraoperative mean arterial pressure and heart rate did not exceed 20% of the basic value. Thirty minutes before the end of the operation, 5 mg tropisetron was given, connected with PCIA, and postoperative analgesia was performed. The formula of the analgesic pump was Sufentanil 2 μg/kg, tropisetron 5 mg, diluted to 150 mL with normal saline, load 2 mL, no background dose, single pressing dose 2 mL, locking time 10 min, maximum dose 12 mL within one hour. In the postanesthesia Recovery unit (PACU) or ward, the analgesic pump PCIA was applied once when the patient actively asked for analgesia or the VAS score was ≥4 after surgery. The pain of the patient was evaluated 15 min later. If the VAS score was still ≥4, intravenous injection of desoxine 5 mg was given for analgesia, and the number of patients with remedial analgesia was recorded.

### 2.3 Observation indicators

Age, height, weight, ASA grading and operation time of the three groups; the resting, coughing and turning VAS scores of the 3 groups at 2 h, 6 h, 12 h, 24 h and 48 h after surgery; the use of sufentanil within 24 h after surgery, duration of QLB block and analgesia (the time from the first press on the analgesic pump after surgery), QoR 40 score 1 day before and 3 days after surgery, occurrence of adverse reactions during perioperative period such as hypotension, bradycardia, PONV, POD, skin itching, shivering, and lower limb movement block were all observed and recorded.

### 2.4 Sample size calculation

This was a randomized controlled trial with VAS score 24 h after surgery as the main outcome index. Sample size calculations based on 10 subjects per group were required to achieve a power of 90% with a type 1 error of 0.05. According to the data obtained from previous studies, VAS score of patients in the three groups at cough or turning over 24 h after surgery were 3.72 ± 1.09 points in group A, 2.69 ± 0.65 points in group B and 2.74 ± 0.73 points in group C. The minimum sample size of each group was 25 cases by PASS 15 software. In consideration of possible dropout, we enrolled 31 subjects per group.

### 2.5 Randomization and blinding

Patients eligible for inclusion were randomly divided into three groups A, B and C by random number table method, with 31 patients in each group. Patient randomized sequences were hidden in sealed opaque envelopes which were only opened by persons who were not involved in this study. The staff prepared same syringes which contained the drugs required by each group. The group situation could not be known from the appearance. The operator of nerve block, the statisticians and the subjects were not aware of the grouping status.

### 2.6 Statistical analysis

SPSS 23.0 statistical software was used for statistical processing. Measurement data were expressed as mean ± standard deviation (x̅ ± s). Single factor analysis of variance was used for inter group comparison, and the Bonferroni test was used for multiple comparisons of time points in the primary analysis. Count data were expressed as frequency or percentage, and comparison between groups was tested by chi-square. *p* < 0.05 was considered statistically significant. Statistical graphs were drawn using GraphPad Prism 8 software.

## 3 Results

### 3.1 Comparison of general data

**Figure 1:**
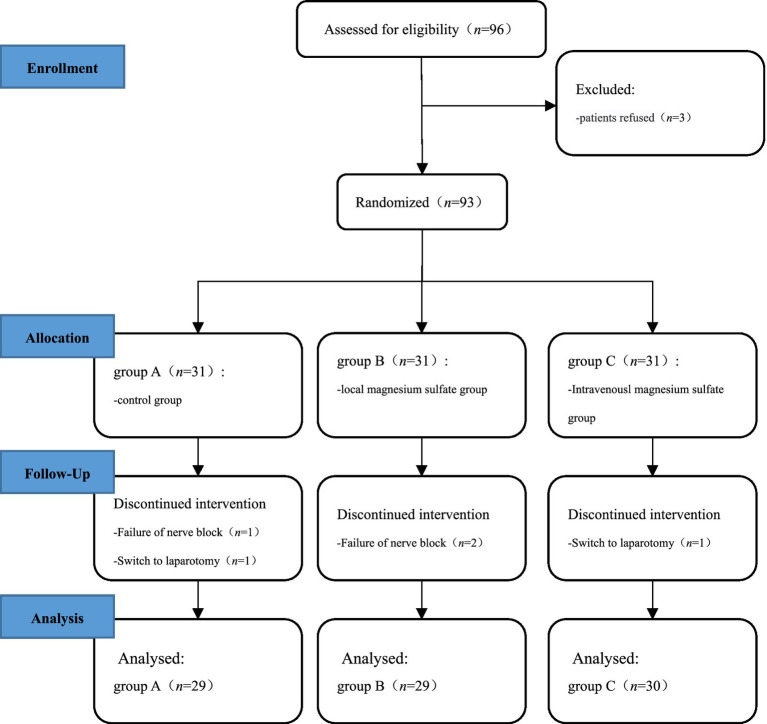
Technology roadmap.

A total of 93 patients undergoing laparoscopic total hysterectomy with general anesthesia were selected. Among them, 3 patients withdrew due to QLB failure, and 2 patients withdrew midway due to the change of intraoperative surgical method. Finally, a total of 88 patients successfully completed the study, including 29 cases in group A, 29 cases in group B, and 30 cases in group C (Figure 1). There were no statistically significant differences in age, height, weight, ASA grading and operation time among the three groups (*p* > 0.05) (Table 1).

**Table 1:** Comparison of general conditions of patients (x̅ ± s).

Group	Age (age)	Height (cm)	Body weight (kg)	Operation time (min)	ASA classification (*n*)	
					II	III
Group A (*n* = 29)	49.28 ± 4.55	156.24 ± 4.75	58.53 ± 6.17	125.14 ± 43.63	20	9
Group B (*n* = 29)	50.31 ± 5.41	155.34 ± 6.24	61.00 ± 7.60	123.31 ± 33.94	16	13
Group C (*n* = 30)	50.13 ± 5.10	156.57 ± 3.29	58.29 ± 6.06	124.50 ± 32.21	22	8
*F/x^2^*	0.351	0.490	1.489	0.018	2.344	
*P*	0.705	0.614	0.231	0.982	0.310	

### 3.2 Comparison of postoperative VAS scores

**Figure 2:**
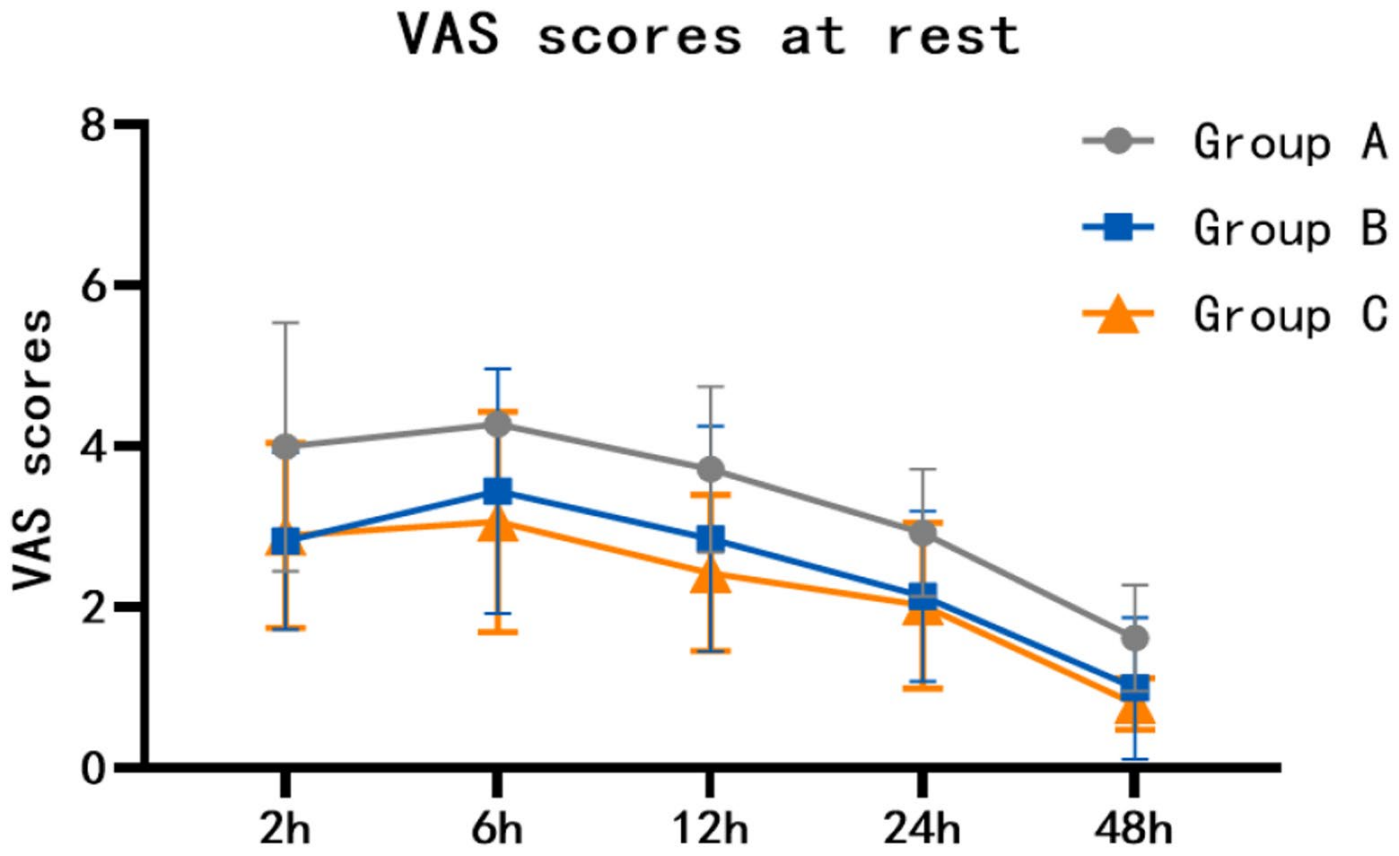
Comparison of resting VAS scores (mean with SD) among the three groups.

**Figure 3:**
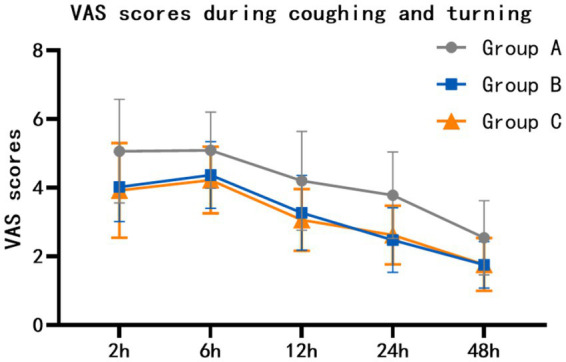
Comparison of VAS scores (mean with SD) during coughing and turning over among the three groups.

At rest, the VAS scores of group B at 2 h, 6 h, 12 h, 24 h and 48 h after surgery were lower than those of group A (*p* = 0.002, *p* = 0.003, *p* = 0.016, *p* = 0.008, *p* = 0.005). The VAS scores of group C at 2 h, 6 h, 12 h, 24 h and 48 h after surgery were lower than those of group A (*p* = 0.004, *p* = 0.001, *p* < 0.001, *p* = 0.002, *p* < 0.001). There was no statistically significant difference between group B and group C (*p* = 1.000, *p* = 1.000, *p* = 0.469, *p* = 1.000, *p* = 0.893) (Table 2; Figure 2). When coughing and turning over, VAS scores of group B were lower than those of group A at 2 h, 6 h, 12 h, 24 h and 48 h after surgery (*p* = 0.011, *p* = 0.024, *p* = 0.010, *p* < 0.001, *p* = 0.001). The VAS scores of group C at 2 h, 6 h, 12 h, 24 h and 48 h after surgery were lower than those of group A (*p* = 0.004, *p* = 0.006, *p* = 0.001, *p* < 0.001, *p* = 0.002). There was no statistically significant difference between group B and group C (*p* = 1.000, *p* = 1.000, *p* = 1.000, *p* = 1.000, *p* = 1.000) (Table 3; Figure 3).

**Table 2:** Comparison of VAS scores at rest [points (x̅ ± s)].

Group	2 h after operation	6 h after surgery	12 h after surgery	24 h after surgery	48 h after surgery
Group A (*n* = 29)	4.00 ± 1.54	4.28 ± 0.09	3.72 ± 1.03	2.93 ± 0.79	1.62 ± 0.66
Group B (*n* = 29)	2.83 ± 1.10^*^	3.45 ± 1.52^*^	2.86 ± 1.40^*^	2.14 ± 1.06^*^	1.00 ± 0.88^*^
Group C (*n* = 30)	2.90 ± 1.15^*^	3.07 ± 1.20^*^	2.43 ± 0.97^*^	2.03 ± 1.03^*^	0.80 ± 0.66^*^
*F*	7.70	6.77	9.583	7.45	10.00
*P*	< 0.001	0.002	< 0.001	0.001	< 0.001

Compared with group A, ^*^P < 0.05.

**Table 3:** Comparison of VAS scores when patients cough and turn over [points (x̅ ± s)].

Group	2 h after operation	6 h after surgery	12 h after surgery	24 h after surgery	48 h after surgery
Group A (*n* = 29)	5.07 ± 1.51	5.10 ± 1.11	4.21 ± 1.44	3.79 ± 1.26	2.55 ± 1.08
Group B (*n* = 29)	4.03 ± 1.01^*^	4.38 ± 0.97^*^	3.28 ± 1.09^*^	2.48 ± 0.94^*^	1.76 ± 0.68^*^
Group C (*n* = 30)	3.93 ± 1.38^*^	4.23 ± 0.97^*^	3.07 ± 0.90^*^	2.63 ± 0.85^*^	1.77 ± 0.77^*^
*F*	6.596	6.076	7.874	13.984	8.058
*P*	0.002	0.003	0.001	< 0.001	0.001

Compared with group A, ^*^P < 0.05.

### 3.3 Comparison of drug usage in postoperative analgesia

Compared with group A, the amount of sufentanil and the number of postoperative rescue analgesia in group B and group C were decreased 24 h after surgery (*p* = 0.036, *p* = 1.000), and there was no difference between group B and group C. Compared with group A, QLB block analgesia duration in group B and group C were prolonged (*p* < 0.001, *p* < 0.001), and there was no difference between group B and group C (*p* = 1.000) (Table 4).

**Table 4:** Comparison of postoperative analgesia and QoR-40 score of patients (x̅ ± s).

Group	Dosage of sufentanil 24 h after operation (μg)	QLB analgesic duration (min)	Postoperative remedial analgesia (*n*, %)	1 day before surgery QoR-40 (points)	3 days after surgery QoR-40 (points)
Group A (*n* = 29)	58.90 ± 14.60	144.21 ± 30.42	8 (27.59)	192.97 ± 3.44	173.66 ± 8.15
Group B (*n* = 29)	51.59 ± 7.39^*^	248.72 ± 46.90^*^	3^*^(10.34)	193.79 ± 3.47	183.21 ± 4.26^*^
Group C (*n* = 30)	50.43 ± 9.22^*^	243.40 ± 36.18^*^	1^*^(3.33)	193.50 ± 3.87	183.37 ± 3.78^*^
*F/x^2^*	5.252	68.409	7.685	0.394	27.566
*P*	0.007	< 0.001	0.021	0.675	< 0.001

Compared with group A, ^*^P < 0.05.

### 3.4 Comparison of postoperative recovery

There was no significant difference in QoR-40 score 1 day before surgery among the three groups (*p* > 0.05). On the 3 day after surgery, QoR-40 score of group B and group C were higher than that of group A (*p* < 0.001, *p* < 0.001), and there was no difference between group B and group C (*p* = 1.000) (Table 4).

### 3.5 Comparison of adverse reactions

PONV occurred in 9 cases (31.03%) in group A, 3 cases (10.33%) in group B, and 2 cases (6.67%) in group C. The incidence rate of Group B and group C were lower than group A, the difference was statistically significant (*p* < 0.05), but there was no statistically significant difference between group B and group C (*p* > 0.05). No complications such as hypotension, bradycardia, POD, skin pruritus, chills, and lower limb movement block occurred in all patients (Table 5).

**Table 5:** Comparison of adverse reactions in patients (*n*, %).

Group	PONV	POD	Itching	Block of motion	Chills
Group A (*n* = 29)	9 (31.03)	0(0)	0(0)	0(0)	0(0)
Group B (*n* = 29)	3^*^(10.33)	0(0)	0(0)	0(0)	0(0)
Group C (*n* = 30)	2^*^(6.67)	0(0)	0(0)	0(0)	0(0)
*x* ^2^	7.503	–	–	–	–
*P*	0.023	–	–	–	–

Compared with group A, ^*^P < 0.05.

## 4 Discussion

Laparoscopic hysterectomy has become the main surgical method of hysterectomy. Compared with open transabdominal hysterectomy, it can reduce postoperative pain, promote postoperative recovery and shorten the length of hospital stay (17, 18). However, the postoperative pain experienced by total laparoscopic hysterectomy patients is still difficult to avoid, with the incidence of incision and upper abdominal pain reaching 69.4% on the first day after surgery (19). QLB is a recently described fascial plane block in which local anesthesia is injected near the quadrate muscle to block the thoracolumbar nerve (20). This block method has been used in various surgeries. Magnesium sulfate is an NMDA receptor antagonist and voltage-gated calcium channel inhibitor that plays a key role in a variety of physiological processes. Magnesium can be used as an adjunct to enhance the analgesic effect.

The results of this study suggested that VAS scores at all time points after the operation and the dose of sufentanil for postoperative intravenous analgesia were decreased of patients with magnesium sulfate. Also, the duration of postoperative QLB block analgesia was prolonged. Meanwhile, relatively speaking, the incidence of postoperative remedial analgesia has also decreased. A randomized, double-blind clinical trial which conducted by Kelany observed the effects of magnesium sulfate combined with bupivacaine during TAP on 60 female patients undergoing open hysterectomy (21). The results showed that compared with the bupivacaine alone group, the postoperative VAS score in the magnesium sulfate combined bupivacaine group was significantly lower, and the average time of first postoperative analgesia was significantly longer. And the total consumption of morphine in the 24 h after surgery was reduced. In another study of Rana (22), the addition of 150 mg magnesium sulfate to local anesthetics resulted in lower postoperative pain scores in patients undergoing open hysterectomy. In the same way, the need for remedial analgesics was reduced, and duration of TAP block analgesia was prolonged. In a randomized controlled trial, Kahraman and Eroglu (23) founded that intravenous infusion of 65 mg/kg magnesium sulfate with spinal anesthesia could extend the duration of spinal sensory block and reduce pain VAS scores in patients undergoing abdominal hysterectomy. And It did not cause any additional complications. The results of our study are consistent with those studies. It indicates that magnesium sulfate, whether used intravenously or locally as an adjuvant can significantly enhance the analgesic effect of QLB in patients undergoing laparoscopic hysterectomy, reduce the use of postoperative opioids, and extend the duration of analgesia. Probably because magnesium ion inhibits calcium ion flow by blocking central and peripheral NMDA receptors, thereby preventing central and peripheral pain sensitization and reducing the release of catecholamines and neurotransmitters, thus producing analgesic effects. Another potential mechanism may be that magnesium carries a cationic charge so that it can neutralize the negative charge on the nerve membrane, thus affecting channel gate of Na+. Then the cell surface was hyperpolarized and the cell activation threshold has been increased. However, the results of another study showed that TAP block with 50% magnesium sulfate combined with 0.2% ropivacaine did not reduce the pain of patients after hysterectomy, which was different from the results of this study. The possible reasons for the difference in the results of the two studies are the different types of surgery (open surgery and laparoscopic surgery) and block techniques (TAP block and QLB block) (24).

In this study, it was founded that intravenous or local use of magnesium sulfate combined with QLB had no statistical significance on postoperative VAS score, consumption of sufentanil, postoperative QoR-40 score and other indicators in patients undergoing laparoscopic hysterectomy. The results indicated that the use of magnesium sulfate (intravenous or local) did not affect the analgesic effect of QLB. Abo-Zeid et al. (25) founded that there was no significant difference in postoperative sedation score, pain score and morphine requirement between group of MgSO4-bupivacaine Pecs II block and group of iv administration of MgSO4, among patients who underwent breast plastic surgery with general anesthesia. It indicated that the two magnesium sulfate administration methods had similar effects on postoperative pain. The results of this study are consistent with those study. This may because that the NMDA receptor is located in the peripheral end of the afferent nerve fibers of muscle and skin and the central nervous system. Therefore, magnesium sulfate can act on the central or peripheral analgesic effects no matter it is used intravenously or locally. Of course, adjusting the dosage may have a more significant analgesic effect, and further studies are needed to explore the optimal dosage of magnesium sulfate. In addition, we did not measure the concentration of magnesium ions in the serum and lacked objective indicators to prove the differences between the two medication methods. Gong’s et al. study (26) showed that the analgesic effect of intraarticular injection of magnesium sulfate was better than that of intravenous injection and intrasheath injection after arthroscopic knee surgery. This is different from our study, which may be related to the type of surgery, specific anesthesia, block mode and dose. So it need further studies.

QoR-40 score has been widely used in clinical practice in recent years, with high reliability, effectiveness and operability, especially suitable for measuring the quality of postoperative recovery (27). The results of this study showed that magnesium sulfate combined with QLB by different routes could improve the total QoR-40 score 3 days after laparoscopic hysterectomy, but there was no statistical difference between the two administration methods. It indicated that intravenous or local use of magnesium sulfate combined with QLB could promote short-term postoperative recovery of patients. This may be related to the reduction of postoperative opioid use and VAS score by both administration modes. Nausea and vomiting after laparoscopic surgery is an unpleasant and painful experience, and the incidence of PONV can be as high as 70 to 80% in patients with high-risk such as women, smoking, vertigo, and postoperative opioid use (28). In this study, the incidence of PONV was lower in patients undergoing laparoscopic hysterectomy with magnesium sulfate. However, there is no difference between the two administration methods. An analysis of 11 randomized controlled trials of Peng et al. (29) founded that intravenous injection of magnesium sulfate during perioperative orthopedic surgery can reduce postoperative analgesic dosage and thus reduce the occurrence of adverse reactions such as vomiting and nausea. The results of our study are similar, suggesting that magnesium sulfate with QLB can reduce nausea, vomiting and other adverse reactions, and provide patients with a comfortable perioperative experience, as a form of multimodal analgesia.

However, there were some limitations in our study. Firstly, this study lacked a complete blank control group to determine whether these results were related to QLB alone or due to the additional use of magnesium sulfate. Secondly, this study did not test other doses of intravenous or topical magnesium sulfate, and we did not measure serum magnesium sulfate concentration. Further research is needed to investigate the correlation between serum magnesium concentration and its effect on postoperative pain and recovery. In addition, due to the limitation of follow-up time, it may have a certain impact on indicators such as the total score of QoR-40. Finally, this experiment is a single-center study with limited experimental conditions and resources, so some large-sample, multi-center experiments are needed in the future to verify the results of this study.

In conclusion, intravenous or local application of magnesium sulfate combined with QLB can reduce postoperative VAS scores, reduce the use of postoperative analgesic drugs, provide effective analgesia, and improve the quality of postoperative recovery of patients, in patients undergoing laparoscopic hysterectomy with general anesthesia. And it did not cause other adverse reactions. In terms of the administration route of magnesium sulfate, intravenous or local use of magnesium sulfate combined with QLB has similar postoperative analgesic effect and postoperative recovery effect in patients undergoing laparoscopic hysterectomy.

## Data Availability

The original contributions presented in the study are included in the article/supplementary material, further inquiries can be directed to the corresponding authors.
